# Generation of Multipotential NG2 Progenitors From Mouse Embryonic Stem Cell-Derived Neural Stem Cells

**DOI:** 10.3389/fcell.2021.688283

**Published:** 2021-08-24

**Authors:** Masahiro Otsu, Zubair Ahmed, Daniel Fulton

**Affiliations:** Neuroscience and Ophthalmology Research Group, Institute of Inflammation and Ageing, College of Medical and Dental Sciences, University of Birmingham, Birmingham, United Kingdom

**Keywords:** neural stem cell (NSC), embryonic stem (ES) cell, NG2 progenitor, oligodendrocyte precursor cell, oligodendrocyte, oligodendrocyte lineage cells, oligodendrocyte differentiation, multipotency

## Abstract

Embryonic stem cells (ESC) have the potential to generate homogeneous immature cells like stem/progenitor cells, which appear to be difficult to isolate and expand from primary tissue samples. In this study, we developed a simple method to generate homogeneous immature oligodendrocyte (OL) lineage cells from mouse ESC-derived neural stem cell (NSC). NSC converted to NG2^+^/OLIG2^+^double positive progenitors (NOP) after culturing in serum-free media for a week. NOP expressed *Prox1*, but not *Gpr17* gene, highlighting their immature phenotype. Interestingly, FACS analysis revealed that NOP expressed proteins for NG2, but not PDGFRɑ, distinguishing them from primary OL progenitor cells (OPC). Nevertheless, NOP expressed various OL lineage marker genes including *Cspg4*, *Pdgfr*α, *Olig1/2*, and *Sox9/10*, but not *Plp1* genes, and, when cultured in OL differentiation conditions, initiated transcription of *Gpr17* and *Plp1* genes, and expression of PDGFRα proteins, implying that NOP converted into a matured OPC phenotype. Unexpectedly, NOP remained multipotential, being able to differentiate into neurons as well as astrocytes under appropriate conditions. Moreover, NOP-derived OPC myelinated axons with a lower efficiency when compared with primary OPC. Taken together, these data demonstrate that NOP are an intermediate progenitor cell distinguishable from both NSC and primary OPC. Based on this profile, NOP may be useful for modeling mechanisms influencing the earliest stages of oligogenesis, and exploring the cellular and molecular responses of the earliest OL progenitors to conditions that impair myelination in the developing nervous system.

## Introduction

During central nervous system (CNS) development, myelinating oligodendrocytes (OL) are established through the proliferation, migration, and differentiation of oligodendrocyte precursor cells (OPC). Oligodendrogenesis is observed throughout the lifespan, beginning in middle gestation when the CNS is populated by OPC that emerge from olig2^+^ neuroepithelial progenitors of the ventricular zones of the spinal cord and brain (reviewed in Bergles and Richardson, 2016), and continuing after birth and into the adult CNS with OPC generated from progenitors located in the forebrain subventricular zone (SVZ) (Levison and Goldman, 1993; Menn et al., 2006; Tsoa et al., 2014). While the location and timing of OPC emergence is clear, questions remain around the nature of the progenitor populations that produce them, with OPC shown to arise directly from neuroepithelial progenitor cells (NPC)/radial glia (Masahira et al., 2006), or indirectly *via* intermediate progenitors such as the glial restricted precursors (GRP) (reviewed in Martins-Macedo et al., 2021), and perhaps other types of immature neural cells, such as OPC pre-progenitors/pre-OPC (Vitry et al., 1999; Baracskay et al., 2007).

*In vitro* studies of embryonic stem cell (ESC)-derived progenitors could provide a useful tool to explore questions relating to the earliest stages of oligodendrogenesis. Several protocols have been published that describe the generation of oligodendroglial lineage cells from mouse ESC (Glaser et al., 2005, 2007; Neman and de Vellis, 2012). In general, these protocols involve culturing of ESC-derived neural stem cells (NSC) in oligodendrogenic factors such as sonic hedgehog, retinoic acid, FGF-2, and PDGF. However, while these protocols efficiently produce mature OPC and terminally differentiated OLs, they do not resolve the earlier steps of oligodendrogenesis ascribed to intermediate progenitors, where mature markers of OPC, such as PDGFRα, are absent (Rao et al., 1998; Baracskay et al., 2007). Here, we describe a novel protocol capable of rapidly generating homogeneous populations of immature NG2^+^/Olig2^+^ progenitors (NOP) from ESC derived from neural stem spheres (Otsu et al., 2011) that display characteristics of intermediate progenitors. Detailed transcriptional and antigenic profiling, referenced to data from primary OPC, indicate that NOP exhibit features common to NPC and OPC phenotypes, such as NG2 expression (Aguirre et al., 2004; Sánchez-González et al., 2020), with the absence of mature OPC markers such as PDGFRα and GPR17 (reviewed in Lecca et al., 2020). Moreover, NOP can be stimulated to undergo sequential differentiation into PDGFRα^+^/GPR17^+^ OPCs, myelinating OLs, and, under appropriate culture conditions, other neural lineages such as astroglia and neurons, thus identifying NOP as a form of intermediate embryonic progenitor cell. NOP are easily maintained as highly proliferative homogenous cultures; hence, they represent a versatile source of cells with which to explore the cellular and molecular processes affecting the earliest stages of oligodendrogenesis.

## Materials and Methods

### Preparation of NSCs From Mouse Embryonic Stem Cells

All data were entirely derived from *in vitro* studies; hence, live animals were not used in this work. NSCs were generated from mouse ESC by the neural stem sphere protocol as described in previous papers (Otsu et al., 2011). ESC were obtained from C57BL/6J mice bred at the University of Birmingham under project license authority held at the University of Birmingham for breeding and maintenance of mice. Mice were killed by a skilled and competent researcher using humane methods proscribed in Schedule 1 of the United Kingdom Government Animals (Scientific Procedures) Act 1986. Isolated NSCs were plated onto matrigel matrix (BD Bioscience, Oxford, United Kingdom)-coated cell culture dishes and maintained into NSC medium [Neurobasal^TM^ Medium supplemented with 2% B-27 supplement, 1% GlutaMAX, 1% Penicillin-Streptomycin (all from ThermoFisher Scientific, Hemel Hempstead, United Kingdom), and 20 ng/ml recombinant human fibroblast growth factor-2 (FGF-2, R&D Systems, Abingdom, United Kingdom)]. Medium change was performed in every other day. After reaching sub-confluency, the NSC were split into new culture dishes. In some experiments, a neural cell population containing a mix of astrocytes, neurons, and NG2 glia were used as controls. The neural cell population was generated by culturing NSC in NSC medium lacking FGF-2 for 6 days to trigger spontaneous neural differentiation (illustrated in Supplementary Figure 1).

### Generation of OL Lineage Cells From Mouse NSCs

NG2^+^/Olig2^+^ progenitors were generated from the NSCs by culturing in OPC medium [DMEM/F12 mixture medium (Sigma-Aldrich, United Kingdom), 1% N2 supplement, 0.5% B-27 supplement, 1% GlutaMAX, 1% Penicillin-Streptomycin, 20 ng/ml FGF-2, 100 ng/ml recombinant human insulin like growth factor-1 (IGF-1, R&D systems), and 20 ng/ml recombinant human platelet-derived growth factor α chain, homodimer (PDGF-AA, Generon Ltd., Maidenhead, United Kingdom)]. Medium change was performed in every other day. After reaching sub-confluency, the cultured cells were split into new culture dishes. In this study, we defined the cultured cells as NOP after maintaining under this condition for at least 7 days. To promote further oligodendrogenesis, NOP were plated in culture dishes filled with OPC medium. After growing at confluency, culture media was changed to OL medium [DMEM/F12 mixture medium, 1% N2 supplement, 2% B-27 supplement, 1% GlutaMAX, 1% Penicillin-Streptomycin, 100 ng/ml recombinant human IGF-1, 40 ng/ml 3,3′,5-Triiodo-L-thyronine (T3, Sigma-Aldrich), and 3.5 ng/ml Hydrocortisone (Sigma-Aldrich)], and the cells were maintained for 6 days to promote terminal differentiation into the OL lineage (NOP/OL). The cell culture protocols for generating NOP and NOP/OL are illustrated in Supplementary Figure 1.

### Preparation of Primary OL Precursor Cells From Postnatal Mouse Forebrain

Primary oligodendrocyte precursor cells (pOPC) were isolated from mixed glial cells derived from postnatal mouse forebrain using the protocol described by O’Meara et al. (2011). In brief, brains from postnatal mice (P2 or P8 C57BL/6 strain) were used for OPC preparation. Mice were sacrificed by methods approved by Schedule 1 of the Animals (Scientific Procedures) Act 1986. Brains were then dissected under a stereomicroscope before enzymatic digestion with 0.025% Trypsin/EDTA solution at 37°C for 20 min. After adding serum-contained medium [10% Fetal bovine serum (FBS) in high-glucose-contained DMEM (both from ThermoFisher Scientific)] and pipetting with serological pipette, followed by passing through cell strainer (Corning Falcon, 70 μm, ThermoFisher Scientific), dissociated cells were collected by centrifugation. Dissociated cells, which mainly consist of mixed glial cells, were plated into poly-L-lysine-coated culture T25 flasks. Mixed glial cells were maintained in serum-containing medium composed of high-glucose DMEM, 10% FBS, 1% Penicillin-Streptomycin, and 2 μg/ml bovine Insulin (Sigma-Aldrich). After reaching confluency (7–10 days), pOPC were isolated from mixed glial cultures by the shake-off protocol. Cell suspensions obtained after the shake off procedure were maintained in uncoated tissue culture grade dishes for 20 min in a CO_2_ incubator to allow the removal of astrocytes and microglia *via* differential adhesion of these cells. The dish was given a gentle swirl once every 5 min to prevent adhesion of OPC, and after 20 min, non-adhered cells (mostly OPC) were collected by removal and centrifugation of the culture medium. The pellet was then resuspended in pOPC medium (DMEM/F12 mixture medium, 1% N2 supplement, 1% B-27 supplement, 1% GlutaMAX, 1% Penicillin-Streptomycin, 20 ng/ml FGF-2, 100 ng/ml IGF-1, 20 ng/ml PDGF-AA, and 3.5 ng/ml Hydrocortisone). High-purity pOPC were plated onto matrigel matrix-coated dishes. To promote OL differentiation, the pOPC were maintained under the conditions described above.

### Astrocyte and Neuronal Differentiation

Cells were plated on matrigel-matrix-coated coverslips with appropriate culture media supplemented with various growth factors overnight. For promoting astrocyte differentiation (condition A), undifferentiated NSC were plated on the coverslips mentioned above. The culture medium was then replaced with glial cell differentiation medium consisting of DMEM/F12 mixture medium, 1% N2 supplement, 2% B-27 supplements, 1% GlutaMAX, 1% Penicillin-Streptomycin, 20 ng/ml recombinant human bone morphogenetic protein-4 (BMP-4), transcript variant 1 (OriGene Technologies, Rockville, MD, United States), 20 ng/ml recombinant human leukemia inhibitory factor (LIF, Cambridge Bioscience, Cambridge, United Kingdom), and 3.5 ng/ml Hydrocortisone. To induce spontaneous neurogenesis (condition B), NSC were maintained for 6 days after removing FGF-2 and/or PDGF-AA from the culture media. Cultures were maintained under the conditions described above (either condition A or B) for 6 days before coverslips were fixed and processed for immunocytochemistry to detect astrogliosis (GFAP) or neurogenesis (βIII tubulin). The culture protocols for examining astroglial and neuronal fate potentials among NOP and are illustrated in Supplementary Figure 1.

### Phase-Contrast Imaging and Measurement of Cell Diameters

Cells were observed with a Nikon Eclipse TS100 inverted microscope equipped with the phase-contrast optics, and images were captured using Digital CCD camera system (DS-L1 and DS-2Mv) (all from Nikon instruments Europe BV, Eadhoevedorp, Netherlands). In the experiment measuring cell diameters of NSC, NOP, pOPC, and mixed glial cells, cells were collected after enzymatic dissociation. After making cell suspension, followed by applying the suspension to hemocytometer (Sigma-Aldrich), images were captured using the system mentioned above. Cell diameters of each cell were measured with ImageJ software on basis of the acquired images. Median and confidence intervals (5 and 95% percentile) of each cell type was calculated using Prism 6 software (GraphPad Software Inc., La Jolla, CA, United States) and plotted to determine representative cell diameters and the apparent purity of each cell type.

### 5-Bromo-2′-Deoxyuridine (BrdU) Incorporation for Cell Proliferation Analysis

To test cell proliferation, cells were plated in Matrigel-coated coverslips and maintained in appropriate culture media for 6 days, at which time cultures were supplemented with 10 μM BrdU for 24 h. After fixation, proliferating cells were detected by immunocytochemical detection of anti-BrdU as described below.

### Immunocytochemistry

Immunocytochemistry was performed using a standard protocol and the following primary antibodies: mouse monoclonal anti-NESTIN (Rat-401, 1:100; Developmental Studies Hybridoma Bank, IA, United States), rabbit polyclonal anti-SOX2 (ab15830, 1:150; Abcam, United Kingdom), rabbit polyclonal anti-brain lipid binding protein (BLBP; AB9558, 1:500; Chemicon, Hampshire, United Kingdom), mouse monoclonal anti-VIMENTIN (V6630, 1:40; Sigma-Aldrich), rabbit polyclonal anti-NG2 (AB5320, 1:150; Millipore, United Kingdom), rabbit polyclonal anti-OLIG2 (AB9610, 1:200; Millipore, MA, United States), mouse monoclonal anti-OLIG2 (SAB1404798, 1:50; Sigma-Aldrich), goat polyclonal anti-PROX1 (AF2727, 1:15; R&D Systems), mouse monoclonal anti-CNPase (C5922, 1:500; Sigma-Aldrich), mouse monoclonal anti-adenomatous polyposis coli (CC1 antibody, OP80, 1:1,000; Calbiochem, La Jolla, CA, United States), mouse monoclonal anti-glial fibrillary acidic protein (GFAP; MAB360, 1:800; Chemicon, CA, United States), mouse monoclonal anti-βIII tubulin (Tuj1, 1:100; BabCO, CA, United States), rat monoclonal anti-myelin binding protein (MBP; MAB386, 1:200, Chemicon), and chicken polyclonal anti-neurofilament heavy polypeptide (NFH; ab72996, 1:25,000; Abcam). Secondary antibodies raised against the appropriate host species and conjugated with Alexa Fluor 488-, Alexa Fluor 594-, and Alexa Fluor 680 dyes were used to detect primary antibodies (1:500; all from ThermoFisher scientific). Cells were plated on Matrigel matrix-coated coverslips or eight-well chamber slides for these experiments. After removing culture media, and washing with PBS, cells were fixed in 4% paraformaldehyde (Sigma-Aldrich) in phosphate-buffered saline (PBS) at room temperature. After washing with PBS, the fixed cells were incubated with 0.2% Triton X-100 (v/v) in PBS at room temperature for 20 min, except in the case of immunocytochemical staining with O4 antibody, where Triton X-100 was omitted. After blocking with 10% bovine fetal serum (ThermoFisher Scientific) or 10% goat serum (S-1000; Vector Laboratory, Peterborough, United Kingdom) in PBS at room temperature for 1 h, primary antibody reactions were carried out under room temperature for overnight. After washing with PBS, secondary antibodies diluted with PBS were added to coverslips and the coverslips were incubated at room temperature for 1 h. After washing with PBS, nuclei were counterstained with 4’,6’-diamidino-2-phenylindole (DAPI) (Molecular Probes). All coverslips were mounted with Immuno-Mount mounting medium (ThermoFisher Scientific). Fluorescent images were obtained using a differential spinning disk confocal microscope (Revolution DSD, Andor Technology, Belfast, United Kingdom) equipped with a 20 × Air Objective (0.5 N.A.).

### Quantitative Real-Time RT-PCR

Messenger RNA (mRNA) isolated from NSC, NOP, and pOPC using RNeasy mini Kit (Qiagen, Crawley, United Kingdom) was used as template for cDNA synthesis with random hexamers, according to the manufacturer’s protocol (Tetro cDNA Synthesis Kit; Bioline, London, United Kingdom). Quantitative real-time RT-PCR was performed on an iQ5 Real Time PCR detection system (Bio-Rad, Hercules, CA, United States) using SYBR^®^ GREEN PCR Master Mix (Applied Biosystems, ThermoFisher Scientific) and Primer Express^TM^ software (Version 2.0, Applied Biosystems)-designed specific primer sets, which were listed in Table 1. The thermal cycling parameters for PCR amplification consisted of 1 cycle at 50°C for 2 min and 95°C for 10 min, followed by 50 cycles of 95°C for 15 s and 60°C for 1 min. The standard curve method was utilized to estimate the mRNA content of each target gene, and the amount of each mRNA was normalized relative to that of GAPDH mRNA. Using positive controls, all primer pairs sufficiently amplified the target genes. The y-axis of each graph regarding qPCR analysis indicates the relative expression level of each target gene. Statistical analysis was performed using Prism 6 software. Data are presented as means ± standard deviation (SD). Statistical significance was determined by unpaired Student’s *t*-test, and *P*-values were corrected with the Sidak-Bonferroni method for multiple comparisons.

**TABLE 1:** List of primers for qPCR.

Gene name	NCBI accession no.	Forward/reverse	Sequence
*Gapdh*	NM_008084.3	Forward	CTGCACCACCAACTGCTTAGC
		Reverse	CAGTCTTCTGGGTGGCAGTGA
*Nes*	NM_016701.3	Forward	AGTGCCCAGTTCTACTGGTGTCC
		Reverse	CCTCTAAAATAGAGTGGTGAGGGTTGA
*Hes1*	NM_008235.2	Forward	ACGGCCAATTTGCCTTTCT
		Reverse	GGAAGGTGACACTGCGTTAGG
*Vim*	NM_011701.4	Forward	CAGCATGTCCAGATCGATGTG
		Reverse	AGCCTCAGAGAGGTCAGCAAAC
*Nkx2-2*	NM_010919.2	Forward	TCAGTCAAGGACATCTTGGACCT
		Reverse	TTCGCTCTCCTCCTCTGGC
*Olig1*	NM_016968.4	Forward	AGGCAGCCACCTATCTCCTCA
		Reverse	AGCGGAGCTTCGGCCTT
*Olig2*	NM_016967.2	Forward	GGCTTCAAGTCATCTTCCTCCA
		Reverse	TCATCTGCTTCTTGTCTTTCTTGGT
*Cspg4*	NM_139001.2	Forward	GAACGCATCAGCCACCGTAA
		Reverse	GGACGCTTCTTCCTGGTTTC
*Pdgfr*α	NM_011058.3	Forward	CCATCGAGACAGGTTCCAGTAGT
		Reverse	GGTCCGAGGAATCTATACCAATGT
*Sox9*	NM_011448.4	Forward	GAAAGACCACCCCGATTACAAG
		Reverse	GGAGAGATGTGAGTCTGTTCCGT
*Sox10*	NM_011437.1	Forward	TCACGACCCCAGTTTGACTATTC
		Reverse	CCCCATGTAAGAAAAGGCTGAA
*Ascl1*	NM_008553.5	Forward	TCCTGTCGCCCACCATCT
		Reverse	TGGGCTAAGAGGGTCGTAGGAT
*Gpr17*	NM_001025381.2	Forward	TCTGGACTTCATCCTCGCTTTT
		Reverse	GGTGCATGAGGAAGACATTGG
*Plp1*	NM_011123.4	Forward	TGGCACTGTTCTGTGGATGTG
		Reverse	GTCCTGGTAGTTTTTGGAGAAATAGGT
*Gfap*	NM_010277.3	Forward	CGTTAAGCTAGCCCTGGACATC
		Reverse	GGATCTGGAGGTTGGAGAAAGTC
*Map2*	NM_008632.2	Forward	AAAGGCCCGCGTAGATCAC
		Reverse	GGGATTCGAGCAGGTTGATG
*Prox1*	NM_008937.3	Forward	TGCCATGAATCCCCAAGGT
		Reverse	GTACGTTCGACTTTTCCCCATCT

### FACS

Cell suspensions for FACS analysis were prepared as followed. After culturing under optimized conditions, the culture medium was removed, and cells were washed with Dulbecco’s Phosphate Buffered Saline (D-PBS, Invitrogen) before harvesting from the culture dish by cell scrapers. After dissociating by pipetting properly, followed by passing through cell strainers to remove chunks, dissociated cells were collected by centrifugation. After removing supernatant, cell pellets were resuspended with cooled FACS buffer [0.5% (v/v) Bovine serum albumin (BSA, Sigma Aldrich), 0.1% NaN3 in D-PBS] supplemented with 1% (v/v) Rat serum for blocking non-specific reaction of antibodies. After incubation at 4°C, cells were pelleted at 300 × g for 5 min and re-suspended in cooled FACS buffer supplemented with Anti-NG2 (Anti-AN2-PE, Clone: 1E6.4, dilution ratio: 1:11, Miltenyi Biotec Ltd., Surrey, United Kingdom), Anti-PDGFRα antibody (CD140a-APC, Clone: APA5, dilution ratio: 1:50, Miltenyi Biotec), and DAPI for counterstaining nuclei (NucBlue^TM^ Live ReadyProbes^TM^ Reagent; Invitrogen, Thermofisher Scientific). After washing with cooled FACS buffer twice before acquisition on a flow cytometer, labeled cell suspension was applied to FACS analysis. The cells expressing each marker protein were counted by BD LSRFortessa^TM^ (BD Biosciences, San Jose, CA, United States) after setting up appropriate gates. All observations were made in log mode. Isotype controls were performed for each primary antibody to ensure the absence of non-specific binding. Data were acquired by using BD FACSDiva^TM^ (BD Biosciences) and analyzed by using Flowing software 2.5.1 (provided by Perttu Terho, Centre for Biotechnology, Turku University, Turku, Finland).

### Myelinating Dorsal Root Ganglion Neuron Co-cultures

For myelination assays, mouse primary dorsal root ganglion neurons (DRGNs) were prepared from postnatal mouse spinal cords following a modified published protocol (O’Meara et al., 2011). In brief, spinal cords from postnatal mice (P2 to P8 C57BL/6 mice) were dissected under a stereomicroscope after culling humanely. After transferring DRGN from the spinal cords to cooled Neurobasal medium supplemented with 1% B-27 supplement, single cell suspensions were prepared by enzymatic digestion in a series of solutions containing 0.025, 0.045, and 0.0625% Trypsin/EDTA (37°C incubation for 35 min). Digestions were followed by mechanical trituration, after which digested DRGN were resuspended with serum-containing medium to stop the enzymatic reaction. Cell suspensions were then passed through a cell strainer (70 μm pore size) and centrifuged, and the resulting cell pellets were resuspended with DRGN medium [1:1 mixture of Neurobasal/2% B-27 and astrocyte-conditioned medium (Sumitomo Bakelite, Tokyo, Japan)] supplemented with 1% Penicillin-Streptomycin and 50 μM cytosine arabinoside (Ara-C, Sigma Aldrich). Dissociated DRGNs were plated at a density of 10,000 cells/well of Matrigel-coated eight-well chamber slides. The DRGNs were maintained for at least 1 week with changing half of medium every other day. For myelination assays, NSCs/NOP/pOPCs were dissociated with Accutase (Invitrogen, ThermoFisher scientific). After centrifugation, followed by removing supernatant, dissociated cells were resuspended with the medium (DMEM:F-12 mixture, 1% N2 supplement, 1% B-27, 100 ng/ml IGF-1, 20 ng/ml PDGF-AA, and 3.6 ng/ml Hydrocortisone) and plated at a density of 50,000 cells/well into wells containing DRGNs. After maintaining in a CO_2_ incubator for 2 days, culture medium was changed with co-culture medium [DMEM/F12 mixture medium, 1% N2 supplement, 1% B-27 supplement, 1% GlutaMAX, 1% Penicillin-Streptomycin, 100 ng/ml IGF-1, 40 ng/ml T3, 10 ng/ml recombinant human neurotrophin-3 (NT-3, Sigma Aldrich), and 3.5 ng/ml Hydrocortisone]. Cells were maintained at 37°C/5% CO_2_ for at least 8 days before examining myelination by immunocytochemistry using anti-MBP and anti-NFH antibodies.

## Results

### Generation of Homogeneous NOP Cells From Mouse ESC-Derived NSC

**FIGURE 1:**
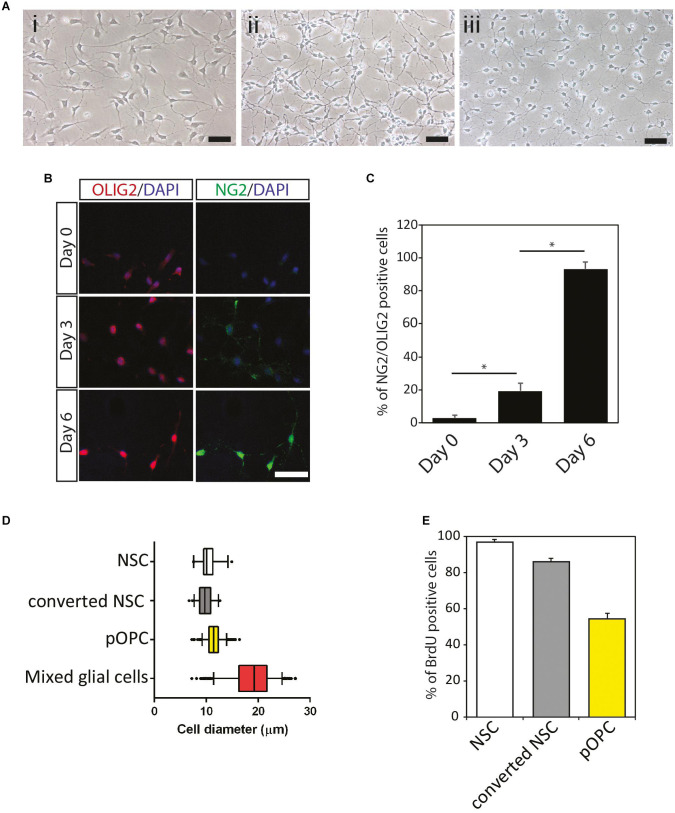
Conversion of mouse neural stem cells to immature NG2^+^/OLIG2^+^ progenitor cells. **(A)** Morphological features of mouse NSC and NSC-derived cells to immature NG2^+^/OLIG2^+^ progenitor cells. Mouse ES cell-derived NSC **(i)**; converted NSC **(ii)**; primary OPC isolated from mixed glial cells **(iii)**. Scale bar: 50 μm. **(B)** Immunocytochemical analysis of oligodendrocyte cell lineage makers NG2 and OLIG2 protein expression in converted NSC. Cells at different culture period (Day 0, 3, and 6) were stained for OLIG2 (red), NG2 (green) and Dapi (blue). Scale bar: 50 μm. **(C)** Percentage of NG2^–^/OLIG2^–^double positive cells during conversion of NSC to OL lineage cell. Data represent means ± SD (bars) of three determinations. *P < 0.05. **(D)** Cell diameter quantification. Boxplots show median and confidence intervals between 5% and 95% (bars) for NSC, converted NSC, pOPC and mixed glial cells. **(E)** Cell proliferation analysis following 24 h BrdU incorporation. Data represent means ± SD (bars) of three determinations. *P < 0.05. n.s., not significant.

Homogeneous and scalable NSC were generated from the ESC using the neural stem sphere method described in previous papers (Figure 1A; Nakayama et al., 2004; Otsu et al., 2011). To generate OL lineage cells from the NSC, NSC were maintained in OPC medium as described in “Materials and Methods” for 7 days (illustrated in Supplementary Figure 1), at which time the morphological features of the cultured cells exhibited subtle changes (Figure 1A). The vast majority of the cells formed bipolar long processes, while NSC tended to display multiple short processes (Figure 1Ai). Moreover, unlike NSC, which exhibited a flattened epithelial-like cell body, cells grown in OPC medium formed rounded cell bodies similar to that of pOPC isolated from mouse forebrain (Figures 1Aii,iii). To determine if these morphological changes were associated with conversion to the OL lineage, we checked expression of representative markers at various time points. Before culturing in OPC medium NSC expressed OLIG2, a pan-OL lineage cell marker, at very low levels, and this expression was mainly localized in the cytoplasm (Figures 1B,C, day 0). Also, NSC expressed very low levels of NG2 protein, a marker associated with OPC (Figures 1B,C, day 0). After 3 days in OPC medium, approximately 20% of the cultured cells expressed both NG2 and OLIG2 proteins (Figures 1B,C). At day 6, over 90% of the cells started to express these markers at high levels (Figures 1B,C). Based on this result, we conclude that maintenance of NSC in OPC medium converted NSC to an NG2/OLIG2-double positive phenotype (converted NSC). To further check the biological properties of the converted NSC, we measured cell diameter, which is a simple parameter for analyzing the purity of cell populations *in vitro*. Similar to NSC and pOPC, converted NSC had a narrow range for the distribution of cell diameter (median approximately 10 μm; Figure 1D). In contrast, mixed glial cultures, which mainly consist of astrocytes with large cell diameters, showed a large distribution for cell diameter with a greater median (approximately 19 μm), implying that converted NSC were maintained as a homogeneous cell population lacking astrocytes (Figure 1D). We also analyzed BrdU incorporation to check the proliferative state of these cells. After 6 days in culture, over 85% of the converted NSC incorporated BrdU, a figure that was comparable to that of NSC (Figure 1E). In contrast, only 50% of pOPC were labeled with BrdU under this condition (Figure 1E); thus, converted NSC were more proliferative than pOPC. Based on their expression of NG2 and OLIG2, and their highly proliferative nature, we define the converted NSC as NOP.

### NSC-Derived NOP Develop Features Toward the OL Lineage

**FIGURE 2:**
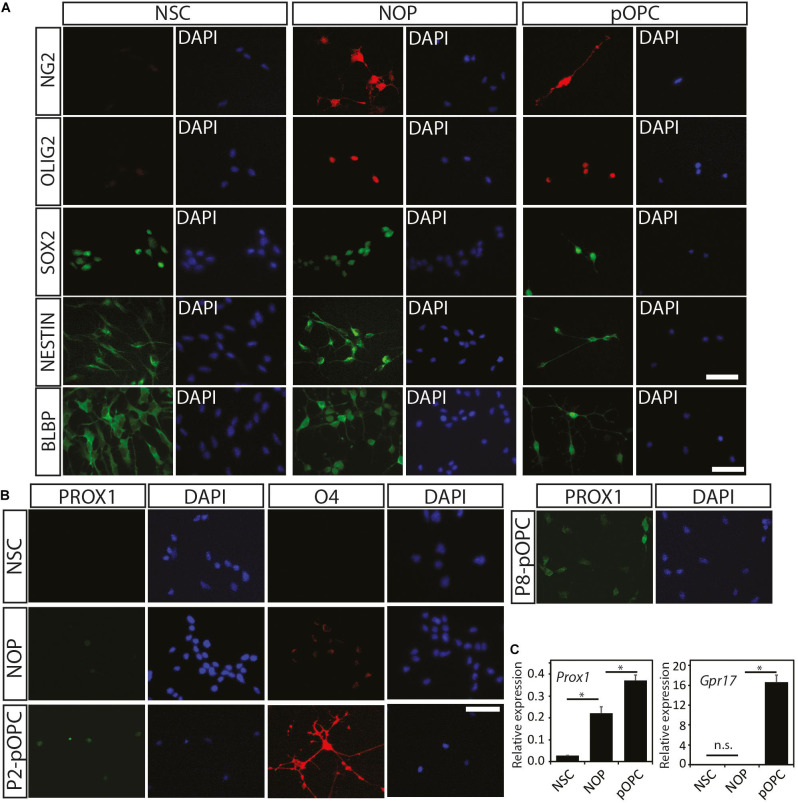
Expression of oligodendroglial lineage markers among NSC, NOP, and pOPC. **(A)** Representative images of immunofluorescent staining for progenitor cell and oligodendroglial lineage marker proteins in NSC, NOP, and pOPC: NG2 (OPC marker); OLIG2 (pan-oligodendrocyte lineage cell marker); SOX2, NESTIN, and BLBP (stem/progenitor cell markers). Scale bar: 50 μm. **(B)** Representative images of PROX1 and O4 signals in NSC, NOP and pOPC (both P2 and P8). Scale bar: 50 μm. **(C)** qPCR based quantification of *Prox1* and *Gpr17* gene expression among NSC, NOP and P2-pOPC. Data are mean ± SD (bars) of three determinations. *P < 0.05; n.s., not significant.

To check whether the biological changes in NOP result from conversion of NSC into OL lineage cells, immunocytochemical analysis was performed with various progenitor and OL lineage progression markers as displayed in Supplementary Figure 2. As described above, NSC expressed OLIG2 and NG2 proteins at very low levels (Figures 1B,C) such that these signals were absent in some imaging fields (c.f. Figures 1B, 2A). In contrast, GFAP (an astroglial cell marker), adenomatous polyposis coli (a marker for differentiated OL detected by antibody clone CC1, henceforth CC1), and MAP2 (a neuronal cell marker) were entirely absent from NSC cultures (data not shown). The absence of these markers for terminal differentiation to neural cells (GFAP, CC1, and MAP2) suggests that homogeneous NSC maintained an undifferentiated state (Figure 2A). On the other hand, NOP, as well as pOPC, expressed NG2 and nuclear-localized OLIG2 protein at high levels, but not the markers for terminally differentiated neural cells (Figure 2A and data not shown). Unexpectedly, the NOP and pOPC expressed NSC-related markers including SOX2, NESTIN, and BLBP (Figure 2A). To explore the degree of OL differentiation in NSC, NOP, and pOPC, we performed immunocytochemistry for anti-O4 and anti-PROX1. O4 is known as a marker for the detection of committed OPC and pre-myelinating OL at early stages of OL differentiation (Gard and Pfeiffer, 1989), while PROX1 is useful for analyzing OL fate determination due to its involvement in the regulation of OL differentiation *via* regulation of notch signaling during oligodendrogenesis (Kato et al., 2011). In this experiment, NSC, which served as a negative control, did not express either the O4-antigen or PROX1 protein (Figure 2B), while NOP expressed these antigens at very low levels (Figure 2B). In contrast, pOPC expressed these proteins at higher levels (Figure 2B). Furthermore, qPCR analysis was performed to confirm the expression of *Prox1* gene, as well as another marker gene, *Gpr17*, which is a useful marker for proliferating OPC and the most immature differentiated OL (Fumagalli et al., 2011; reviewed in Lecca et al., 2020). *Prox1* gene expression was significantly increased in NOP and pOPC in comparison with that in NSC, while *Gpr17* gene was undetectable in NSC and NOP, but robustly expressed in pOPC (Figure 2C). The elevated levels of *Prox1* transcripts and low levels of O4 antigen imply a shift toward the OL lineage, while the absence of *Gpr17* expression indicates a more immature developmental status than pOPC. Data from DRGN-co-culture experiments provide further support for this immature phenotype. Typically, pOPC display a robust capacity to differentiate into myelinating MBP^+^ OL when cultured with DRGN (O’Meara et al., 2011), and indeed, we found that 7 days old DRGN-pOPC co-cultures contained a number of MBP + OL whose MBP + processes contacted and aligned with DRGN axons (Supplementary Figures 3A,B). In marked contrast, MBP^+^ cells were absent in NSC seeded cultures (Supplementary Figure 3C), and extremely rare when DRGN were seeded with NOP, with only a single example found in one coverslip (Supplementary Figure 3D). Overall, the absences of *Gpr17* transcripts, and an apparent inefficiency in converting to MBP^+^ cells, imply that NOP represent a more immature phenotype than fully committed OPC.

### NOP Upregulate PDGFRɑ Protein After T3 Stimulation

**FIGURE 3:**
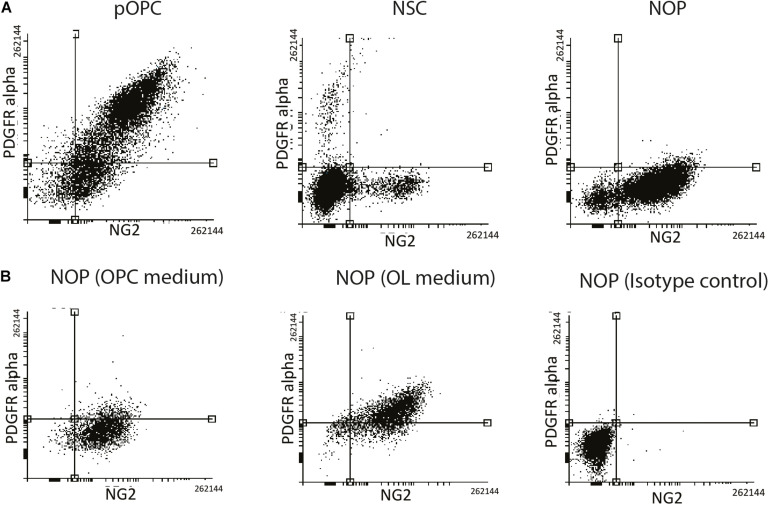
NOP acquire an pOPC-like phenotype after T3 stimulation. **(A)** Absence of PDGFRα protein distinguishes NOP from pOPC. Expression analysis of NG2 (X-axis) and PDGFRα (Y-axis) from cultures of NSC, NOP and pOPC by FACS. **(B)** FACS analysis of NG2 (X-axis) and PDGFRα (Y-axis) signals from NOP, and NOP after a 6-day growth period in OL medium containing T3. Left and middle panels display data from NOP and NOP cultures after OL medium (respectively) that were labelled with anti-NG2 and anti-PDGFRα. Right panel displays negative control data from NOP labelled with isotype control antibodies for each marker protein as a negative control.

In general, NG2 expressing glia are well-known to express the OPC marker protein PDGFRα isoform (PDGFRɑ), whose ligand, PDGF-AA, regulates cell properties including migration and proliferation of OL lineage cells (Richardson et al., 1988; Tognatta et al., 2017). However, there is evidence that expression of NG2 and PDGFRα by OL lineage cells can be variable and unsynchronized during the later stages of oligodendrogenesis (Zuo et al., 2018). To test the hypothesis that NOP and pOPC have different expression patterns of NG2 and PDGFRα, FACS analysis was carried out to quantify the percentage of cells expressing these marker proteins. As expected, over 70% of pOPC were double positive for both proteins, while 15% of pOPC expressed only NG2 protein (Figure 3A, left panel). NSC were analyzed as a negative control showing that only a small proportion of NSC expressed a single marker (Figure 3A, middle panel). In contrast to pOPC, the majority of NOP expressed only NG2 antigen, with PDGFRα expressing cells almost completely absent (Figure 3A, right panel). To explore the properties of NOP further we analyzed their expression of NG2 and PDGFRα antigens after growth in OL medium containing T3 and lacking growth factors. For this experiment, we analyzed NOP 6 days after the transition from OPC to OL medium and compared this to NOP maintained in OPC medium. Consistent with the previous data, NOP maintained in OPC medium expressed antigens for NG2, but not PDGFRα (Figure 3B, left panel). However, after growth in OL medium, over 70% of the NOP population were double positive for NG2 and PDGFRα, while approximately 20% of them expressed only the NG2 antigen (Figure 3B, middle panel). This profile is similar to that of pOPC (Figure 3A); thus, growth in OL medium stimulates the conversion of NOP to an NG2^+^/PDGFRα^+^ phenotype resembling that of neonatal-derived pOPC. The specificity of these profiles was confirmed by analysing NOP labeled with appropriate isotype control antibodies (Figure 3B, right panel). Taken together, these *in vitro* data identify the absence of PDGFRα antigens as a key characteristic distinguishing NOP from pOPC, and demonstrate that NOP exhibit the potential for oligogenesis by converting to an OPC-like phenotype after growth in OL medium.

### NOP Undergo Oligogenesis and OL Differentiation After T3 Stimulation

**FIGURE 4:**
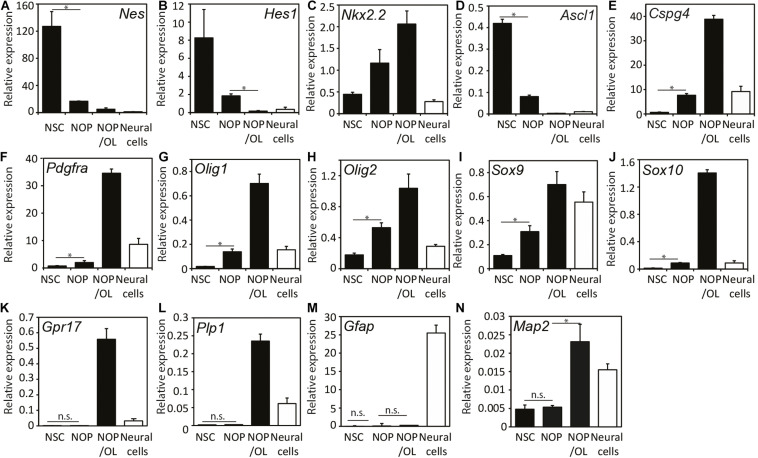
Expression patterns of progenitor and neural lineage marker genes during oligogenesis from neural stem cells. Expression analysis of various genes including NSC-markers *Nes* **(A)** and *Hes1* **(B)**, region-specific transcription factors *Nkx2.2* **(C)** and *Ascl1* **(D)**, OPC-markers *Cspg4* **(E)** and *Pdgfa* **(F)**, OL-lineage cell markers *Olig1* **(G)**, *Olig2* **(H)**, *Sox9* **(I)**, and *Sox10* **(J)**, OPC/pre-OL marker *Gpr17* **(K)**, OL marker *Plp1* **(L)**, astrocyte marker *Gfap* **(M)**, and neuronal marker *Map2* **(N)**. mRNAs were isolated from the cells after culturing in either OPC medium for NOP, or OL medium for differentiated NOP/OL, and relative gene expression was analyzed using quantitative real-time qPCR. Expression levels of each mRNA were normalized relative to that of GAPDH mRNA. Black bars indicate the expression level in three different population (NSC, NOP, and NOP/OL, and white bars show the expression levels in neural cells derived from NSC as control. Data are mean ± SD (bars) of three determinations. *P < 0.05; n.s., not significant.

To further explore the capacity of NOP for oligogenesis and OL differentiation, we examined the expression of various NSC and OL lineage genes (see Supplementary Figure 2) in four different cell populations: NSC, NOP, NOP cultured in OL medium for 7 days (NOP/OL), plus NSC-derived neural cells (see Supplementary Figure 1), which contain astrocytes, neurons, and NG2-expressing cells (Supplementary Figure 4). NSC markers like *Hes1*, *Nestin* (*Nes*), and *Ascl1* genes were markedly downregulated in NOP and hardly expressed in NOP/OL (Figures 4A–D). The decrease in NSC maker expression in NOP confirms that NSC are converted toward a more restricted progenitor phenotype following growth in the OPC medium. Indeed, the expression of OPC marker genes like *Cspg4* and *Pdgfr*α genes was increased 10 and 2.5 times, respectively, in comparison to levels in NSC. Importantly, these markers, along with *Olig1/2*, *Nkx2.2*, and*Sox9/10*, were further upregulated in the NOP/OL population (Figures 4E–J), indicating progressive oligogenesis when NOP are cultured in OL medium. Consistent with the progression of oligogenesis, differentiation markers *Grp17* and *Plp1*, which were absent in NSC and NOP, appeared in the NOP/OL population (Figures 4K,L). On the other hand, *Gfap*, an astrocyte marker, was hardly expressed during this oligogenesis (Figure 4M), but was expressed strongly in neural cells, which contain an abundance of astrocytes (Figure 4M and Supplementary Figure 4B). Unexpectedly, OL medium caused NOP to upregulate *Map2* mRNA (Figure 4N). However, the relative expression for this gene (normalized to GAPDH) was markedly lower than that of all OL related genes. For example, expression levels of MAP2 were approximately 10-fold lower than *Plp1* (MAP2, 0.023; *Plp1*, 0.23), the OL gene with the lowest relative expression level. Overall, these gene expression data, and the antigen profiling shown in Figure 3, show that NOP undergo oligogenesis when cultured in T3 containing OL medium.

To validate these gene expression data, and confirm the presence of mature OL in the NOP/OL population, we performed immunocytochemistry for protein markers of committed OPC and mature OL. After culturing in OL medium for 7 days, NOP/OL showed clear expression of antigens detected by anti-O4, anti-CNPase, and anti-CC1 (Supplementary Figures 5A–C, respectively). Quantification of this immunostaining revealed that 32.8% (±2.03) of DAPI^+^ cells expressed the O4 antigen, a marker for committed OPC (Gard and Pfeiffer, 1989), while a smaller fraction were immuno-positive for the mature OL markers CNPase^+^ (19.94 ± 2.93%) and CC1 (19.53 ± 3.07%) (Supplementary Figure 5D). Overall, the gene and protein expression data presented above provide clear evidence that NOP undergo progressive oligogenesis and OL differentiation when grown under conditions designed to stimulate OL differentiation.

### NOP Differentiate Into Mature OL With a Lower Efficiency Than pOPC

**FIGURE 5:**
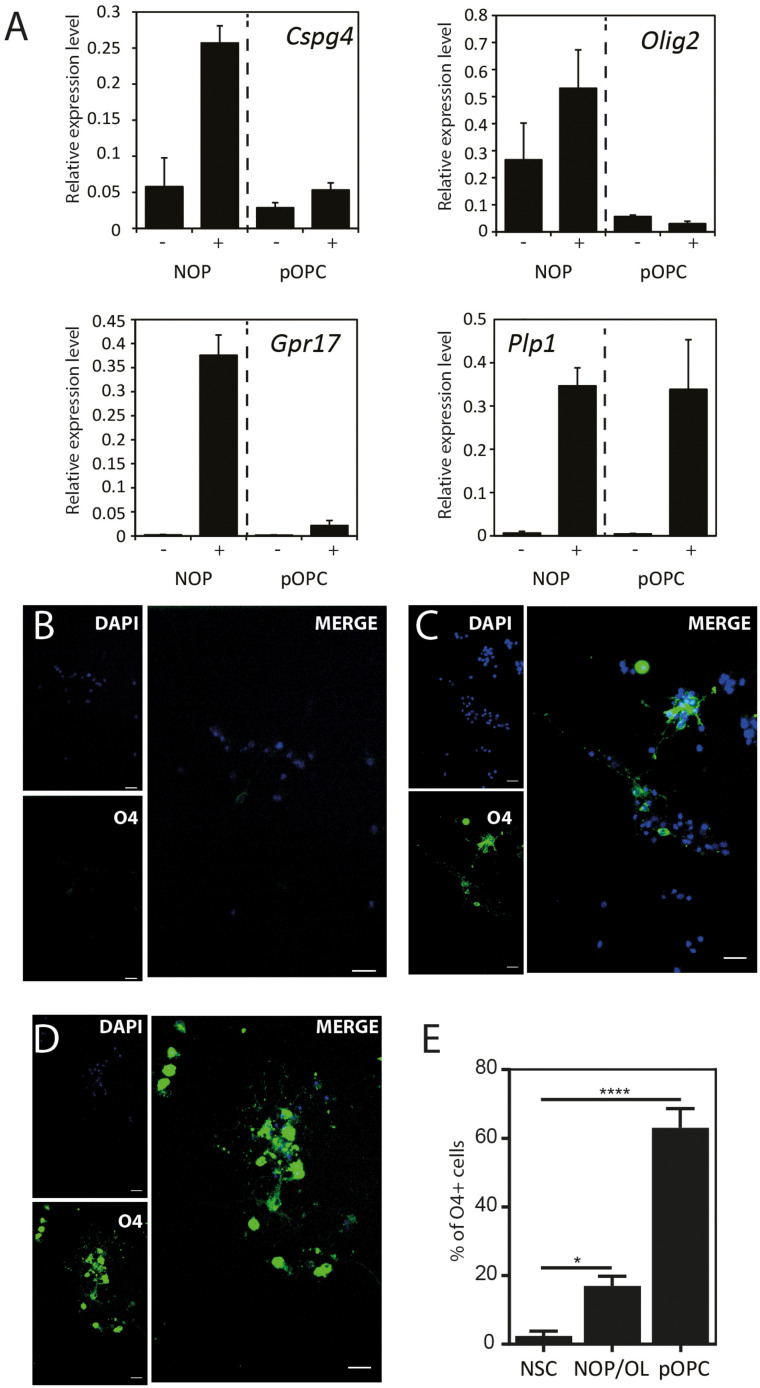
NOP differentiate into OL with a lower efficiency than pOPC. **(A)** Quantification of OL lineage-related genes after culturing in OL differentiation medium: OPC-marker (*Cspg4*); OL-lineage-related transcription factor (*Olig2*); OPC/pre-OL marker (*Gpr17*); OL marker (*Plp1*). mRNA were isolated from NOP or pOPC either before or after culturing in OL differentiation medium for 6 days, and relative gene expression analyzed by qPCR. Expression levels of each mRNA were normalized relative to that of GAPDH mRNA. Graphs indicate expression levels from four cell populations: NOP, T3-stimulated NOP (NOP/OL), pOPC, and differentiated pOPC (pOPC/OL). Data are mean ± SD (bars) of three determinations. **(B–D)** Representative images of immunofluorescent staining for O4 antigen in NSC **(B)**, NOP **(C)**, and pOPC **(D)** after culturing in OL differentiation medium. Nuclei were counterstained with DAPI. Scale bar: 20μm. **(E)** Percentage of O4 antigen positive OL in cultures of NOP and pOPC after culture in OL differentiation medium. Data represent means ± SD (bars) of three determinations. *P < 0.05; ****P < 0.001.

**FIGURE 6:**
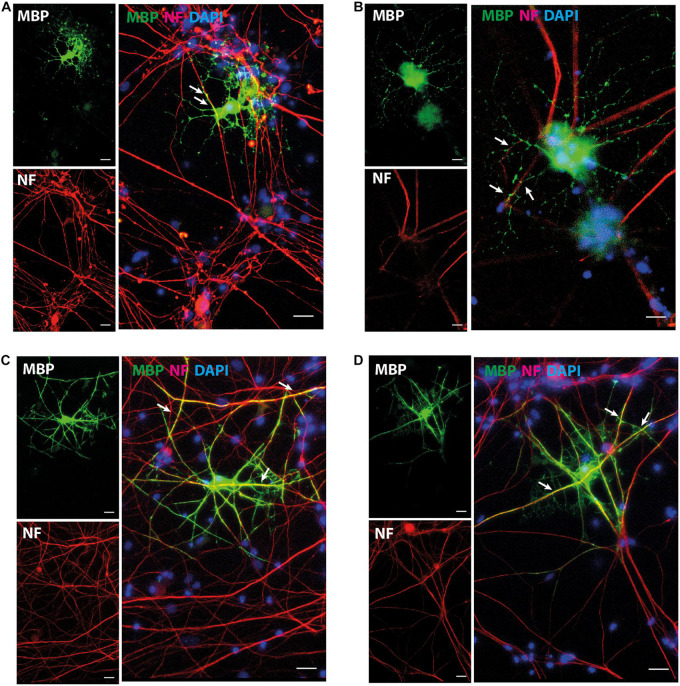
NOP/OL initiate axonal contact in DRGN co-cultures. Representative images of immunofluorescent staining for myelin basic protein (MBP, myelin) and neurofilament heavy-chain polypeptide (NF, axons) in DRGN cultures seeded with NOP/OL **(A,B)** and pOPC **(C,D)**. After 7 days in co-culture, some NOP/OL have differentiated into MBP^+^ OL whose processes contact DRGN axons. Contacts appear as fine MBP + process aligned with short stretches of NF^+^ axon (**A,B**, white arrows). pOPC show more advanced signs of myelination, with MBP^+^ processes forming longer stretches of axonal alignment (**C,D**, white arrows, yellow profiles). Nuclei were counterstained with DAPI. Scale bar: 20 μm.

The expression pattern for mature OL marker proteins after growth in OL medium (between approximately 20 and 30%, Supplementary Figure 5) indicates that the process of OL differentiation was rather slow in the NOP population. To explore this question, we compared the expression of genes associated with OL lineage progression and differentiation (*Cspg4*, *Olig2*, *Gpr17*, and *Plp1*) in NOP and pOPC before and after growth in OL medium. Although the expression profiles of NOP and pOPC were similar before this treatment, differences in the levels *Cspg4* and *Gpr17* levels appeared following this stimulation (Figure 5A). Interestingly, despite the increased levels of these genes in the NOP/OL population, the levels of the myelin gene *Plp1*, a marker for terminal OL differentiation, were similar across NOP and OPC populations after T3 stimulation. In line with this expression of *Plp1*, the NOP/OL population also expressed transcripts for myelination components such as *Mbp*, *Mog, Mag*, and Gap junction genes (*Gjb1*, *Gjc2*, and *Gjc3*) when cultured in the presence of DRGN axons (Supplementary Figure 6); thus, NOP/OL clearly present a transcriptional profile expected of mature differentiated OL. To determine if these transcriptional profiles translate into a mature antigenic phenotype, we measured the fraction of differentiating O4^+^ OL in NSC, NOP, and pOPC populations after culturing in OL medium for 7 days. Whereas NSC hardly differentiated into O4^+^cells (Figure 5B), both NOP and pOPC clearly differentiated into O4^+^ OL lineage cells (Figures 5C,D). However, NOP converted to O4^+^ cells with less efficiency than pOPC (Figure 5E), confirming that OL differentiation was less efficient in the NOP/OL population. Finally, to confirm the potential of NOP to complete terminal differentiation into mature OL, we examined MBP^+^ profiles in DRGN cultures seeded with either NOP/OL or pOPC. In contrast to NOP-DRGN cultures, which virtually lacked MBP^+^ OL (Supplementary Figure 3), co-cultures seeded with NOP/OL contained a number of MBP^+^ OL whose processes showed signs of early axonal contact (Figures 6A,B). Of note, these contacts are reminiscent of the spiraling of fine MBP^+^ processes previously identified during the early stages of *in vitro* myelination (Ioannidou et al., 2012). Interestingly, these contacts differ to those observed in DRGN cultures seeded with pOPC, where MBP^+^ processes extended long segments that aligned with DRGN axons (Figures 6C,D). Taken together, these data demonstrate that the NOP/OL population contains cells that have undergone terminal differentiation into mature OL, that these OL present the early signs of myelination, and that these steps toward myelination appear to be less advanced than those achieved by pOPC.

### NOP Maintain a Potential to Differentiate Into Astrocytes and Neurons

**FIGURE 7:**
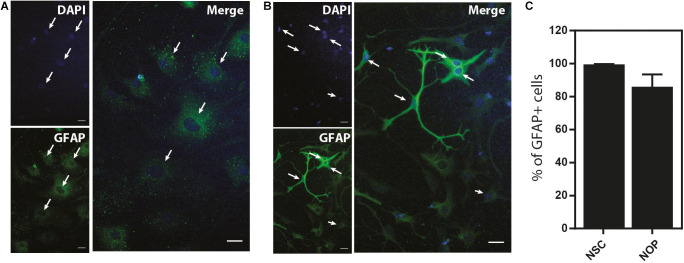
Neural stem cell and NOP convert into GFAP^+^ astrocyte-like cells. Representative images of immunofluorescent staining for GFAP protein in NSC **(A)** and NOP **(B)** after stimulation with human BMP-4 and LIF to promote astrocyte differentiation. Nuclei were counterstained with DAPI. White arrows indicate examples of DAPI^+^/GFAP^+^ profiles. Scale bar: 20 μm. **(C)** Percentage of DAPI^+^/GFAP^+^ cells after BMP-4 and LIF treatment of NSC and NOP. Data represent means ± SD (bars) of three determinations.

**FIGURE 8:**
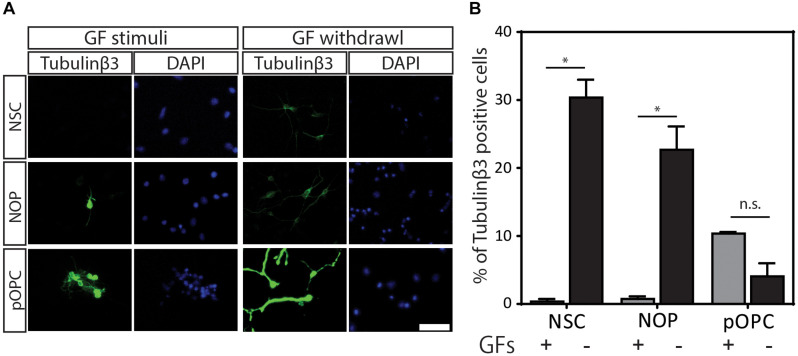
Neurogenic potential of NOP. **(A)** Representative images of immunofluorescent staining for neuronal marker protein ßIII-tubulin protein in NSC, NOP, and pOPC after culturing in growth factor-depleted basal medium to promote spontaneous neuronal differentiation. Nuclei were counterstained with DAPI. Tubulinβ3: βIII-tubulin protein; Scale bar: 50 μm. **(B)** Percentage of βIII-tubulin-positive cells after culture of NSC, NOP, and pOPC in conditions promoting neurogenesis. Data represent means ± SD (bars) of three determinations. *P < 0.05; n.s., not significant.

Our data show that NOP resemble early progenitors. We therefore determined whether they retained an NSC-like multipotentiality by examining their ability to generate astrocytes and neurons. We compared the potential for astrogenesis among NSC and NOP by culturing these cells in BMP4/LIF-containing medium for 6 days. Following this treatment, NSC converted to flattened GFAP^+^ cells similar to those observed in primary cultures of mouse astrocytes (Schildge et al., 2013; Figure 7A). In contrast, NOP differentiated into GFAP^+^ cells displaying a mix of morphologies including flattened GFAP^+^ cells, and cells with multiple thickened GFAP^+^ processes typical of ESC derived astrocytes (Juneja et al., 2020; Figure 7B). Consistent with previous reports (Suzuki et al., 2017), we also detected GFAP + cells when cultures of pOPC were treated with BMP4 and LIF (data not shown). Quantification of the proportion of GFAP^+^ cells in NSC and NOP cultures revealed similar numbers (Figure 7C), implying that NSC and NOP have a similar potential to differentiate into astrocyte-like cells. Next, we examined the potential for neurogenesis among NSC, NOP, and additionally pOPC by culturing them in growth factor depleted medium. Prior to this treatment, we noted the presence of a small number of βIII tubulin^+^ primary neurons contaminating the population of pOPC (approximately 10%), whereas these cells were virtually absent in cultures of NSC and NOP (approximately 1%; Figure 8A). After culturing in growth factor-depleted medium to promote spontaneous differentiation, both NSC and NOP differentiated into neurons at a comparable rate (approximately 30% in NSC cultures; 22% in NOP cultures, Figure 8B), while the total percentage of neurons in pOPC cultures declined during this period (Figure 8B). Thus, under these conditions NOP retain the potential to convert to neurons, while pOPC appeared to be restricted to a glial phenotype.

## Discussion

In this study, we established a protocol for rapidly generating high-purity cultures of immature NG2 progenitor cells from mouse ESC-derived NSC. These cells, which we term NOP, exhibit an antigenic profile consistent with an immature neural/oligogenic progenitor phenotype (e.g., nuclear OLIG2^+^, NG2^+^, GFAP^–^, CC1^–^, and MAP2^–^), and under differentiating conditions, these cells sequentially convert into OPC expressing both NG2 and PDGFRα antigens, and mature OL capable of initiating the early stages of axon myelination. Immature astrocytes also express OLIG2 proteins during CNS development (Marshall et al., 2005). However, NG2 proteins, which are well established as a marker of OPC, are not characteristic of astrocytes (reviewed in Nishiyama et al., 2005). Thus, taken together, the upregulation of transcripts for *Pdgfr*α and *Olig1/2*, and the absence of GFAP protein support the positioning of NOP within the oligodendroglial lineage. Despite this profile, the typical OPC marker protein PDGFRα is absent from NOP and is only expressed after further maturation is encouraged by T3 stimulation and growth factor withdrawal. Moreover, NOP retain marker proteins for NSC like SOX2, NESTIN, and BLBP, and are multipotential, generating astrocytes and neurons when cultured under appropriate conditions. Overall, NOP appear to be an immature progenitor intermediate between NSC and fate-restricted OPC whose rapid generation into scalable high-purity cultures recommends them for further studies to explore fundamental properties of early gliogenesis and neurogenesis.

NOP were small cells with a simple bipolar morphology. This morphology is similar to that of pOPC derived from developing mouse and rat CNS tissues (Chen et al., 2007) and of gliogenic progenitors of the SVZ (Levison and Goldman, 1993), but contrasts with the morphology of mature parenchymal OPC/NG2-glia observed *in vivo*, and cultured brain slices, which display a complex array of fine branching processes (Butt et al., 2005; Wigley et al., 2007; Fulton et al., 2010; Fannon et al., 2015). Notably, the morphology of NOP is similar to that of multipotential postnatal SVZ progenitors (Levison and Goldman, 1997); thus, the simple morphology of NOP is consistent with an intermediate progenitor phenotype. In line with this classification, markers associated with OPC differentiation, such as *Prox1* (Kato et al., 2011), *Gpr17* (Fumagalli et al., 2011), and O4 antigen (Gard and Pfeiffer, 1989), were barely detectable in NOP, but were upregulated strongly in response to growth factor withdrawal and treatment with T3, a stimuli typically used to trigger OPC differentiation *in vitro*. NOP also lacked expression of PDGFRα, a protein with well-defined roles in promoting OPC proliferation (Pringle et al., 1989) and survival (Barres et al., 1992). This antigenic profile differed markedly to that of pOPC, which we confirmed to express high levels of PDGFRα, PROX1, O4 antigen, plus mRNA for *Gpr17*. NOP therefore displayed morphological, antigenic, and functional characteristics of immature intermediate progenitors.

The OPC medium used to convert NSC to the early-NG2 glia contained PDGF-AA and IGF-1, yet NOP lacked PDGFRα. IGF-1 exerts influences over both NSC and OPC (reviewed in Ziegler et al., 2015); thus, it is likely that the conversion process was stimulated by IGF-1. Culturing of NOP in OL medium lacking PDGF-AA and containing T3 stimulated the expression of PDGFRα protein. The timing of PDGFRα expression in NOP differs to that observed in the embryonic nervous system, where PDGFRα expression precedes that of NG2 by 2 days (Nishiyama et al., 1996). Similarly, a recent single-cell transcriptomic analysis of human OPC report distinct peaks for PDGFRα and NG2 expression, with NG2 peaking later in development than PDGFRα (Perlman et al., 2020). However, the developmental profile of PDGFRα transcripts we observe before and after differentiation of NOP (Figure 3) is consistent with data from human fetal cells where A2B5^+^ glial precursors exhibit markedly lower levels of these transcripts compared to committed OPC (Leong et al., 2014). Similarly, analysis of mixed CNS cultures isolated from various postnatal CNS regions revealed a population of NG2^+^ progenitors that lacked expression of the A2B5, a defined marker for OPC *in vitro* (Baracskay et al., 2007). Interestingly, analysis of embryonic cortical explants indicates the presence of NG2^+^ progenitors prior to the arrival of A2B5^+^ progenitors, while *in vitro* studies identify them as direct ancestors to A2B5^+^ OPC. Based on these observations, Baracskay et al. (2007) propose a model that, in good agreement with the present findings, places NOP as an *in vitro* pre-progenitor to OPC.

In keeping with an immature progenitor phenotype, NOP express transcripts associated with NSC such as *Nes*, *Hes1*, and *Ascl1*, and are tripotential, exhibiting a capacity to generate O4^+^/MBP^+^ OL, and astrocyte-like GFAP^+^ cells and βIII tubulin^+^ neurons when cultured in appropriate conditions. The NOP/OL population downregulates these NSC genes during differentiation in OL medium; thus, it would be interesting to compare their astroglial and neuronal potential with the more immature NOP cells. Regarding the identify of NOP-derived GFAP+ cells, BMP4 stimulation, as used here, is recognized to drive the differentiation of NPC toward an astroglial fate (Gross et al., 1996); thus, the GFAP+ cells, which had morphologies expected of cultured mouse astrocytes (Schildge et al., 2013; Juneja et al., 2020), are likely to represent astroglial lineage cells. Nevertheless, GFAP is also expressed by NPC/radial glia populations; thus, in the absence of other lineage markers, we term these cells astroglial-like. The astroglial potential of NOP is consistent with a number of genetic fate mapping studies, revealing the production of astrocyte populations from embryonic NG2-glia (Zhu et al., 2008a,b, 2011; Huang et al., 2014, 2019) and NG2-glia/OPC *in vitro* (Diers-Fenger et al., 2001; Suzuki et al., 2017). However, the extent and anatomical distribution of this astrogenesis remains an open question, with some studies reporting NG2-glia derived astrogenesis in the spinal cord, cerebellum, and ventral forebrain (Zhu et al., 2008a,b), and others either reporting a distribution restricted to ventral forebrain alone (Huang et al., 2019), or finding no direct evidence to support this fate (Tognatta et al., 2017). Nevertheless, the astrocytic potential of NOP is reminiscent of GRP, a distinct progenitor stage connecting NSC to OL and astrocyte lineages that express *Nes*, and that can be isolated from embryonic spinal cord tissues (reviewed in Martins-Macedo et al., 2021). However, as implied by their name, GRP lack neurogenic potential. In contrast to this, a neurogenic fate has been discussed for NG2-glia. For example, NG2-glia expressing a CNPase-GFP transgene were found to generate electrically active hippocampal neurons *in vivo* (Belachew et al., 2003). Of note, the same study identified a population of NESTIN^+^/NG2^+^ glia within the SVZ of early postnatal mice, implying a link between this antigenic profile, which we also describe for NOP, and a neurogenic potential. Similarly, a neurogenic potential among NG2-glia has been demonstrated by *in vitro* fate mapping of retrovirus-labeled SVZ progenitors (Levison and Goldman, 1997), and *in vivo* by genetic fate mapping methods (Rivers et al., 2008; Guo et al., 2010). However, other studies using different reporter lines have failed to confirm these observations (Zhu et al., 2008a; Huang et al., 2014) and the neurogenic potential of NG2-glia remains controversial (reviewed in Guo et al., 2021). Notwithstanding this debate, other OPC genes, such as proteolipid protein (PLP), are associated with a neurogenic progenitor phenotype during embryonic development (Delaunay et al., 2008). Interestingly, both embryonic *Plp1*-progenitors and NOP express the radial glia marker protein BLBP, and a recent genetic fate mapping study has linked transient NG2 expression to embryonic radial glia (Tognatta et al., 2017). Taken together, these data identify oligodendroglial gene expression as a feature that marks certain populations of embryonic neurogenic progenitors. In this context, NOP represent a promising tool with which to investigate the neurogenic potential of embryonic NG2-progenitors. In particular, studies examining the fate of NOP following CNS transplantation would provide a test of the neurogenic potential of embryonic NG2-glia that could complement current data from cre recombinase-based fate mapping studies (reviewed in Guo et al., 2021). Moreover, irrespective of the existence, or not, of a neuronal fate among endogenous NG2-glia, the potential benefits of cell-based neurogenic therapies provide a strong motivation to explore the fates of NOP *in vivo*.

In our protocol, NOP were generated from mouse ES cells within 18 days, with a further 7 days required to convert these cells to a typical OPC phenotype expressing PDGFRα, O4, and Gpr17 antigens. The generation of OPC/OL from ES cells within 25 days is considerably faster than other protocols using mouse ES cells and other non-CNS sources. For example, (Neman and de Vellis, 2012) report a protocol generating mature OL from mouse ES cells within 48 days, while Zhang et al. (2019) generated functional OL from bone marrow derived OPC within 40–50 days. The relatively short protocol, and the ability to easily isolate homogeneous populations of NOP, represents a useful feature of the present protocol. Moreover, NOP are highly proliferative; thus, they can be easily expanded to provide large numbers of cells to support a range of applications, including further studies to explore the fundamental properties of embryonic NG2-glia/OL pre-progenitors (Baracskay et al., 2007), including their *in vivo* fate potential, or to enable the rapid production of OPC for conventional *in vitro* assays involving OL differentiation and myelination, such as the screening of pro-myelination compounds.

In conclusion, the new protocol we have described provides a rapid and efficient means to produce homogenous populations of immature ES-derived NOP that can be sequentially converted to populations of OPC and myelinating OL, or terminally differentiated into other types of neural cells such as astrocyte and neurons. Using this system, we have derived data that identifies NG2 as marker for an intermediate OL pre-progenitor that lacks the complete antigenic profile for OPC. Therefore, in addition to providing an efficient and scalable source of OPC/OL for *in vitro* studies of myelination, we predict this method could be used to further advance our knowledge of NG2 glial functions, and examine their involvement in the earliest stages of oligodendrogenesis.

## Data Availability Statement

The raw data supporting the conclusions of this article will be made available by the corresponding author upon reasonable request.

## Ethics Statement

The animal study was reviewed and approved by University of Birmingham Animal Welfare and Ethical Review Board.

## Author Contributions

MO and DF concept and design of the study. MO cell culture and generation of NOP and data acquisition and analysis. MO, ZA, and DF drafting of manuscript and figures. All authors read and approved the submitted version of the manuscript.

## Conflict of Interest

The authors declare that the research was conducted in the absence of any commercial or financial relationships that could be construed as a potential conflict of interest.

## Publisher’s Note

All claims expressed in this article are solely those of the authors and do not necessarily represent those of their affiliated organizations, or those of the publisher, the editors and the reviewers. Any product that may be evaluated in this article, or claim that may be made by its manufacturer, is not guaranteed or endorsed by the publisher.
