# Supplementary figures and images for: Generation of Multipotential NG2 Progenitors From Mouse Embryonic Stem Cell-Derived Neural Stem Cells
Source: Front Cell Dev Biol. 2021 Aug 24;9:688283. doi: 10.3389/fcell.2021.688283 (PMC8423355; doi:10.3389/fcell.2021.688283)

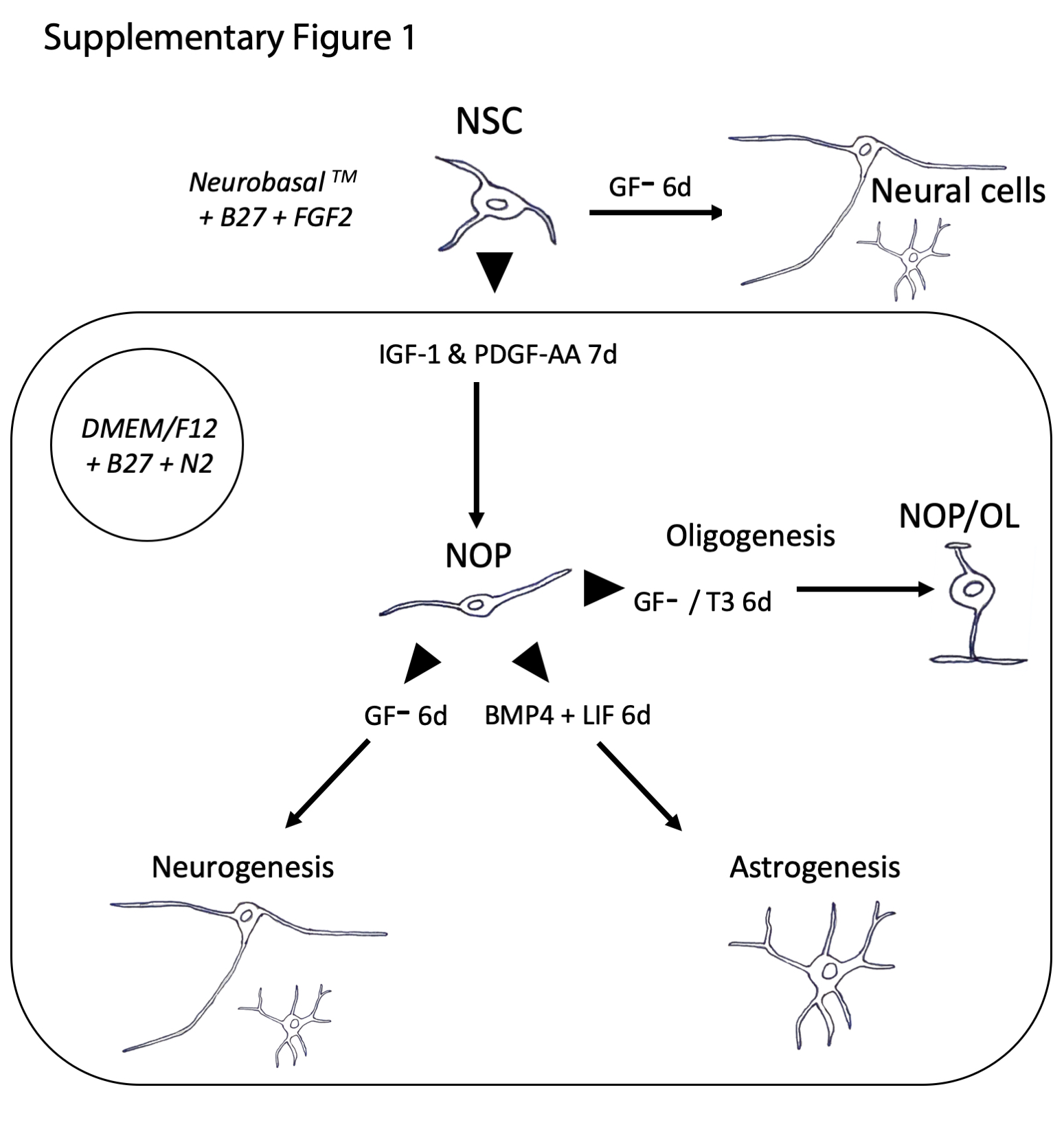

Supplement: Supplementary file 1 [file Image_1.JPEG]

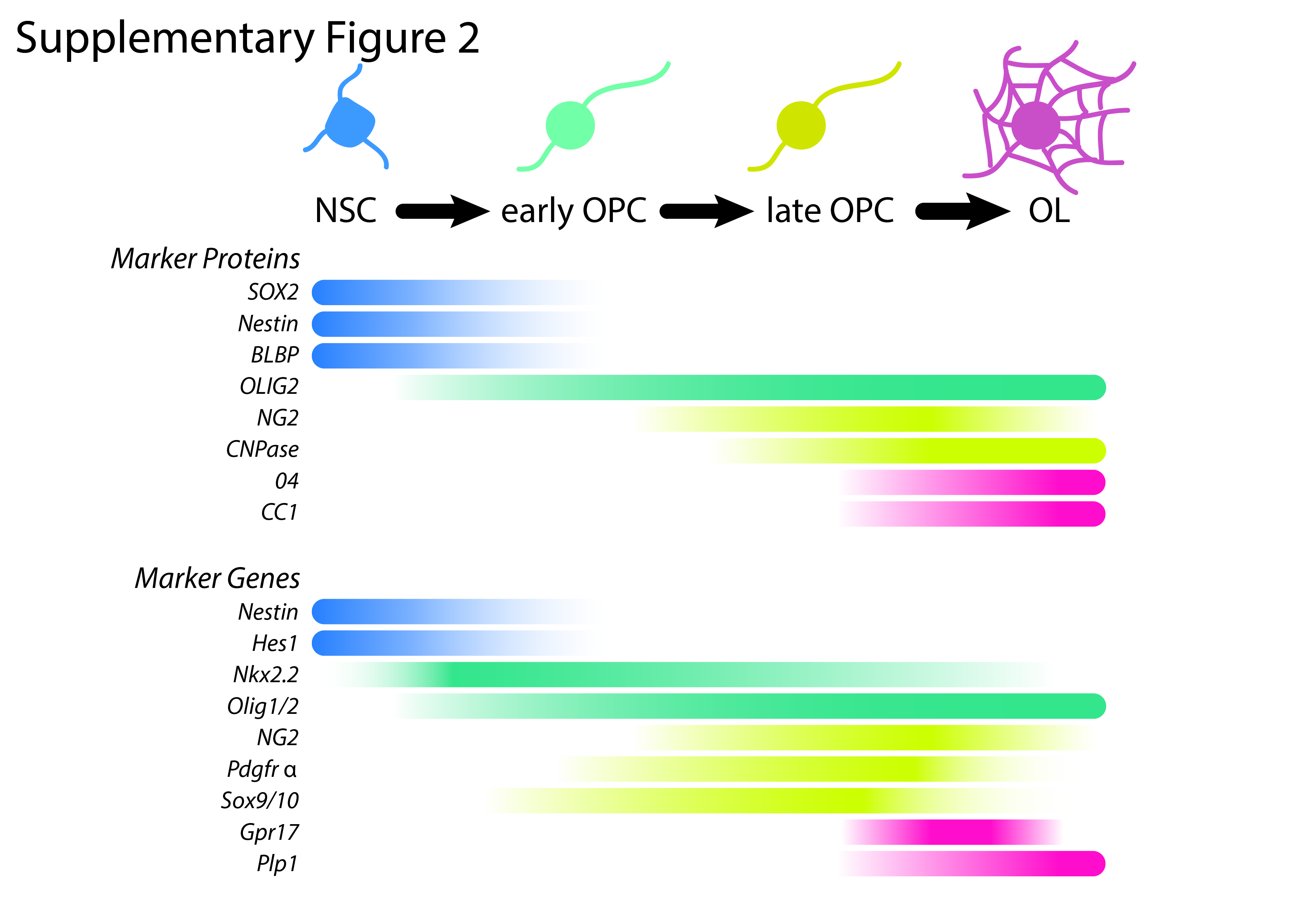

Supplement: Supplementary file 2 [file Image_2.JPEG]

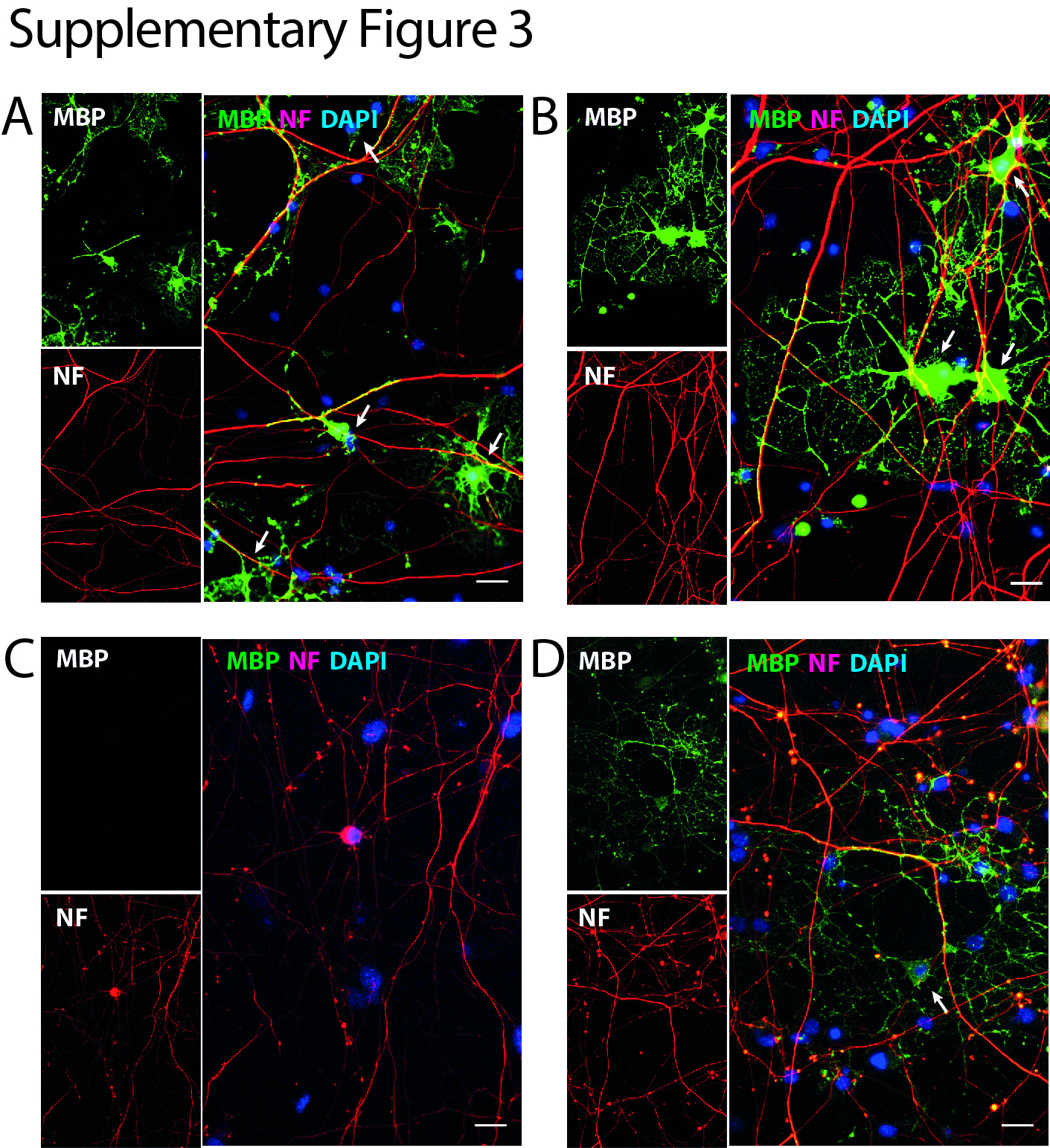

Supplement: Supplementary file 3 [file Image_3.JPEG]

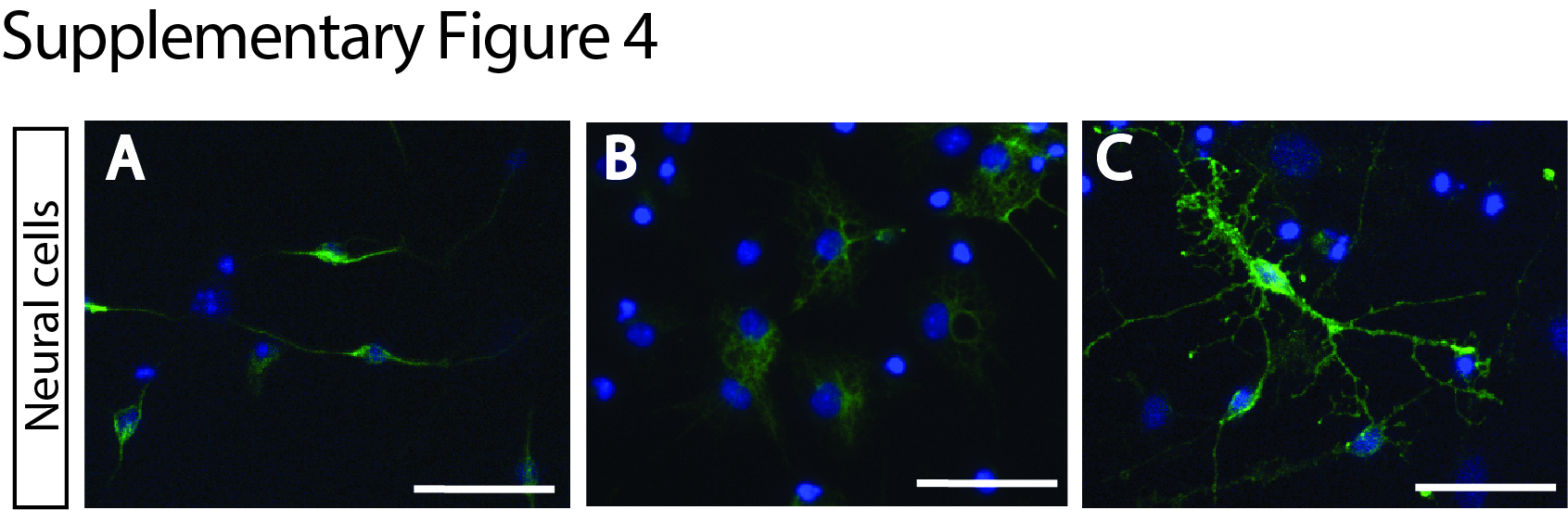

Supplement: Supplementary file 4 [file Image_4.JPEG]

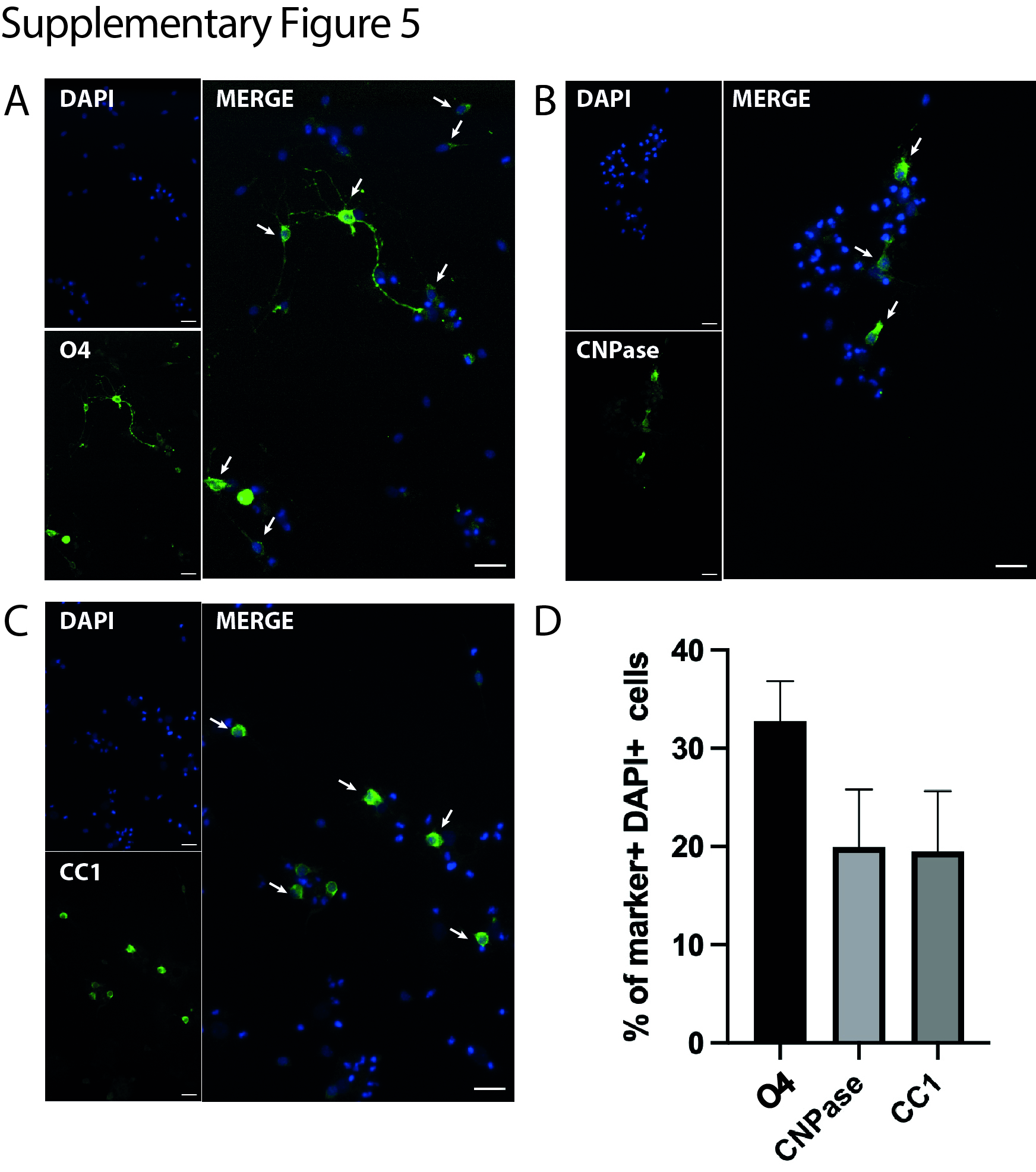

Supplement: Supplementary file 5 [file Image_5.jpg]

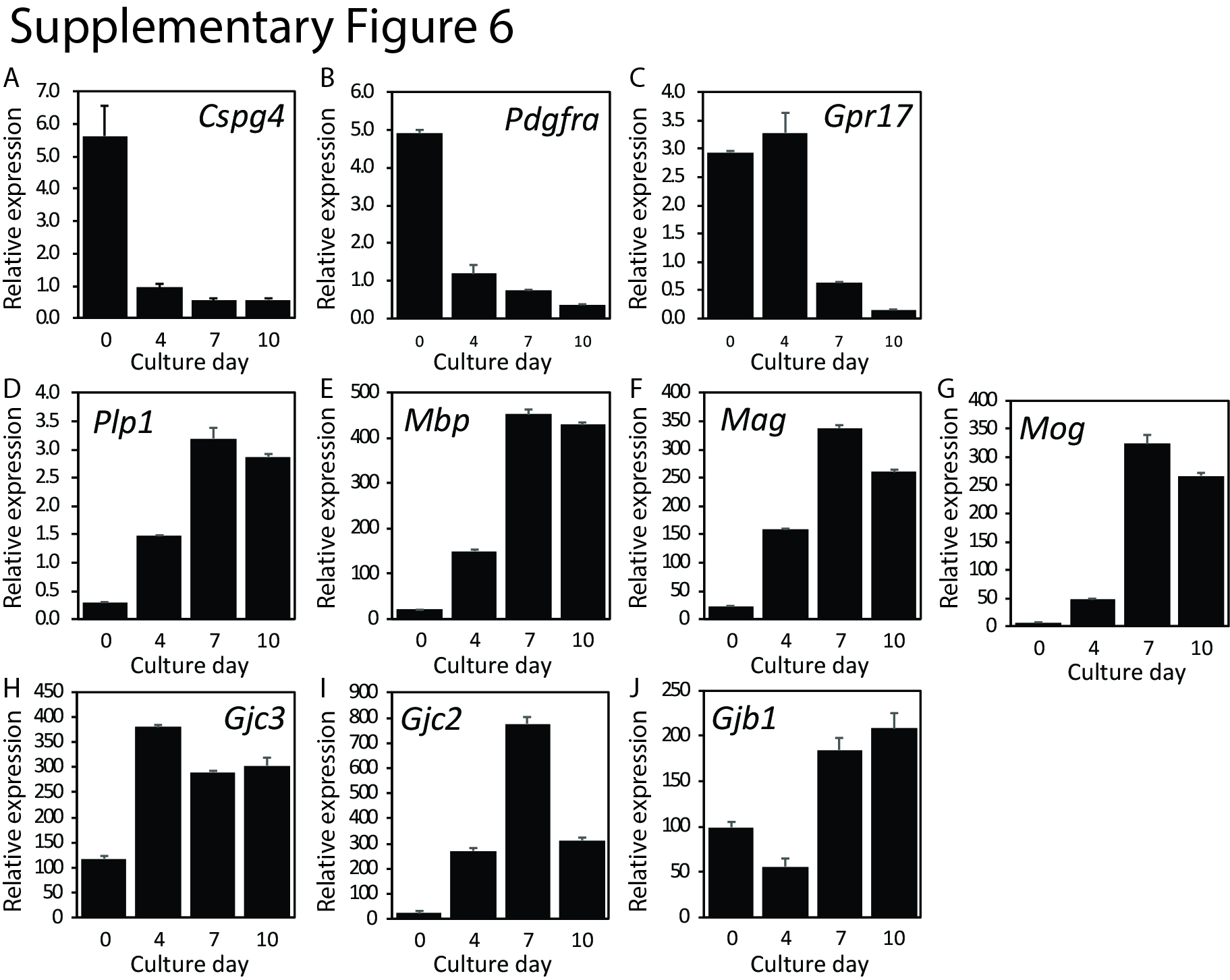

Supplement: Supplementary file 6 [file Image_6.JPEG]
