# Supplementary material for: Generation of Multipotential NG2 Progenitors From Mouse Embryonic Stem Cell-Derived Neural Stem Cells
Source: Front Cell Dev Biol. 2021 Aug 24;9:688283. doi: 10.3389/fcell.2021.688283 (PMC8423355; doi:10.3389/fcell.2021.688283)
Supplement: Supplementary file 7 [file Data_Sheet_1.docx]

**Generation of multipotential NG2 progenitorsfrom mouse embryonic stem cell-derived neural stem cells**

Masahiro Otsu^1,2^, Zubair Ahmed^1^ and Daniel Fulton^1^*

^1^Neuroscience and Ophthalmology Research Group, Institute of Inflammation and Ageing, College of Medical and Dental Sciences, University of Birmingham, Edgbaston, Birmingham B15 2TT, UK. ^2^ present address Braizon Therapeutics, Inc. Kanagawa, Japan. *To whom correspondence should be addressed**.** *Tel*: +44-121-455-48441; *Fax*: +44-1214-148-867*E-mail*: [d.fulton@bham.ac.uk](mailto:d.fulton@bham.ac.uk).

**Keywords:** oligodendrocyte precursor cell, oligodendrocyte, oligodendrocyte lineage cells, embryonic stem cell, neural stem cell, NG2glia, PDGFRα, Gpr17, multipotential

**Supplementary Material**

**Supplementary Figure Legends**

**Supplementary Figure 1. Schematic representation of culture protocols**

Schematic depicting the culture protocols used to generate NOP, NOP-derived cells, and NSC-derived neural cell populations from NSC. NSC are maintained as a homogenous population by culture in medium containing FGF-2. NSC are converted to the neural cell population containing astrocytes, neurons and NG2^+^ cells by culturing in medium lacking FGF-2 for 6 days. NSC are converted to NG2^+^/OLIG2^+^ progenitors (NOP) by culture in OPC medium containing IGF-1 and PDGF-AA for 7 days. NOP grown in OL medium lacking growth factors and supplemented with T3 differentiate into a population of oligodendroglial lineage cells including committed OPC and differentiated OL. NOP undergo neurogenesis when grown in medium lacking growth factors, and can also be converted to astrocyte-like cells by sustained stimulation with BMP4 and LIF.

**Supplementary Figure 2. Schematic representation of oligodendrocyte cell lineage identified by marker proteins and genes during oligogenesis**

Schematic of the oligodendroglial lineage spanning NSC to differentiated oligodendrocyte via early and late OPC, and depicting the stage specific expression patterns for key marker genes and proteins during this process. Colour intensity of each marker shows the degree of expression at each stage.

**Supplementary Figure 3. Imaging pOPC and NOP differentiation in DRGN co-cultures**

Images of immunofluorescent staining for myelin basic protein (MBP, myelin), and neurofilament heavy-chain polypeptide (NF, axons) in DRGN cultures seeded with pOPC (A&B), NSC (C) and NOP (D). White arrows indicate MBP^+^ OL. MBP^+^ OL were easily observed in co-cultures seeded with pOPC (A&B). In these cultures differentiated OL were seen to align MBP^+^ profiles with NF^+^ DRGN axons (yellow profiles). MBP^+^ OL were absent in NSC seeded cultures(C), and scarcely encountered in NOP seeded cultures (D). Note, MBP^+^ OL in panel D exhibits contact with an NF^+^axons consistent with the early stages of myelination. Nuclei were counterstained with DAPI. Scale bar: 20um.

**Supplementary Figure 4. Expression of neural lineage marker proteins in NSC-derived neural cells.**

Expression of neural cell marker proteins Tubulinβ3 (neurons, A), GFAP (astrocytes, B) and NG2 (OPC, C) in NSC-derived neural cells. Scale bar: 50um.

**Supplementary Figure 5. Expression of OL marker proteins in NOP/OL**

Expressionof OL lineagemarker proteins for committed OPC (O4) and differentiated OL (CNPase, CC1)in the NOP following growth in OL medium for 7 days. A-C. Representative images of immunofluorescent staining forO4 (A), CNPase (B), and CC1 (C).Nuclei were counterstained with DAPI.White arrows identify marker^+^/DAPI^+^ profiles. Scale bar: 20µm. D. Histogram displayingquantification of marker^+^/DAPI^+^ profilesas percentages of total DAPI^+^cells. Data are mean± SD (bars) of four determinations.

**Supplementary Figure 6. Expression patterns of myelination-related genes in the NG2/OL population during myelination**

Expression analysis of various myelination-related genes including OPC-markers *Cspg4* (A) and *Pdgfa*(B), OPC/pre-OL marker *Gpr17* (C), OL markers *Plp1* (D)*, Mbp*(E)*, Mag* (F), and *Mog* (G), and Gap junction markers *Gjc3* (H), *Gjc2* (I), and *Gjb1* (J). mRNAs were isolated from NG2/OL after co-culture with DRGN for various periods (0, 4, 7 and 10 days). Relative gene expression was analyzed using quantitative real-time RT-PCR. Expression levels of each mRNA arenormalized relative to that of GAPDH mRNA. Closed bars in each graph indicate the relative expression level of each marker genes among various time points. Data are mean± SD (bars) of three determinations.
